# Petal stomata of *Hemerocallis citrina* Baroni are sensitive to abscisic acid

**DOI:** 10.3389/fpls.2025.1570821

**Published:** 2025-06-17

**Authors:** Lei Gong, Ye Hua, Yun-Yun Su, Bo Zhang, Li-Ting Yao, Basmah M. Alharbi, Md. Mahadi Hasan

**Affiliations:** ^1^ School of Agriculture and Bioengineering, Longdong University, Qingyang, China; ^2^ Gansu Key Laboratory of Protection and Utilization for Biological Resources and Ecological Restoration, Qingyang, China; ^3^ Biology Department, Faculty of Science, University of Tabuk, Tabuk, Saudi Arabia; ^4^ Biodiversity Genomics Unit, Faculty of Science, University of Tabuk, Tabuk, Saudi Arabia; ^5^ State Key Laboratory of Herbage Improvement and Grassland Agro-ecosystems, College of Ecology, Lanzhou University, Lanzhou, China

**Keywords:** petal stomata, floral gas exchange, stomatal aperture, ABA signaling, drought stress adaptation

## Abstract

In response to drought stress, abscisic acid (ABA) plays a crucial role in regulating stomatal closure in both leaf and floral tissues. Studies on stomatal regulation have primarily focused on the leaves of vascular plants, but stomatal regulation of flowers remains underexplored. The current study was conducted on the petals of ‘Ma Lin’ daylily (*Hemerocallis citrina* Baroni) to assess the morphological characteristics of petal stomata, stomatal aperture, gas exchange, and the mechanisms of ABA signaling in response to treatments with ABA-related chemicals and their corresponding scavengers. The study showed that stomata are primarily located in the lower epidermis of the petals, arranged in a strip near the central vein, and exhibit relatively low density; the guard cells contain a large number of chloroplasts. Exogenous ABA induced stomatal closure in the petal stomata, and the gas exchange assay indicated that stomatal conductance decreased when exogenous ABA was introduced into the transpiration stream. The stomatal aperture assay revealed a 32.78% decrease following a 10 µM ABA treatment. Furthermore, both hydrogen peroxide (H_2_O_2_) and nitric oxide (NO) were involved in the ABA-induced stomatal closure process, with H_2_O_2_ acting as an upstream component of NO. Overall, these results suggest that physiologically active stomatal control is present in the flower of ‘Ma Lin’ daylily under drought stress, consistent with the regulation observed in leaf stomata.

## 1 Introduction

Droughts, which are increasingly common worldwide, are expected to intensify under future extreme conditions (Adams et al., 2017; Choat et al., 2018; Bi et al., 2023). This scarcity of precipitation causes severe water shortages in plant tissues, leading to reduced yields in crop species and damage to wild ecosystems (Sack and Tyree, 2005; Blackman et al., 2019; Yao et al., 2021). To mitigate water loss and maintain homeostasis under drought, plants rely heavily on the regulation of stomata (Gong et al., 2025). Stomata are microscopic pores on the leaf surface that regulate gas exchange between the plant and its environment (Hasan et al., 2024). These pores are flanked by guard cells that modulate their opening (stomatal aperture) in response to internal and external cues, thereby controlling water loss and CO_2_ uptake (Hasan et al., 2021). Land plants regulate their stomatal aperture by altering guard cell turgor to survive in adverse climates (Sussmilch et al., 2017a, 2019a). Stomatal conductance, rather than stomatal aperture, on the leaf surface accurately reflects physiological processes as it determines the rate of gas exchange of water vapor and CO_2_ (Ochoa et al., 2024). Consequently, a decline in gas exchange contributes to drought tolerance and plant survival in low precipitation environments (Blackman et al., 2014; López et al., 2021). Over the past decades, it has been established that abscisic acid (ABA) is a key hormone inducing stomatal closure in response to drought across a wide range of seed plants through complex and precise signal transduction processes, including ABA biosynthesis, perception, and activation of multiple ion channels (Mittelheuser and Van Steveninck, 1969; Trejo et al., 1995; Cutler et al., 2010; Raghavendra et al., 2010; Lim et al., 2015; Chen et al., 2020; Hsu et al., 2021).

Recent research has revealed that stomatal responses to ABA are exclusive to seed plants, with species such as ferns and lycophytes not exhibiting ABA-induced stomatal closure, a phenomenon linked to the absence of SnRK2-mediated slow-type anion channel (SLAC) activation (Brodribb and McAdam, 2011; McAdam and Brodribb, 2012, 2014; McAdam et al., 2016a, b; Sussmilch et al., 2017b). This gradualistic theory of stomatal evolution suggests that some species adapted to dry conditions exhibit high stomatal closing efficiency. Meanwhile, recent other studies have suggested that ABA also induces stomatal closure in mosses, lycophytes, and ferns (Chater et al., 2011, 2016; Ruszala et al., 2011; Horak et al., 2017; Cai et al., 2017). The explanations for the contradiction between these two statements regarding ferns and lycophytes could be either different growth conditions or different experimental conditions (Horak et al., 2017; Cai et al., 2017). While the differing stomatal responses among vascular plant lineages have been widely studied and debated (Sussmilch et al., 2019b; Gong et al., 2021), these investigations have primarily focused on the stomata of leaves, with little attention to specialized tissues such as reproductive organs and subterranean bulbs, which may exhibit unique regulatory mechanisms (Sussmilch et al., 2019b).

*Hemerocallis citrina* Baroni (daylily), a perennial herbaceous plant from the Asphodelaceae family, is renowned for its edible flowers, medicinal properties, and ornamental value in horticulture (Lin et al., 2013; Matand et al., 2020; Misiukevičius et al., 2023; Sun et al., 2024). Widely cultivated as a vegetable and medicinal herb for thousands of years in China and Eastern Asia, the daylily is often referred to as the long yellow daylily (LYD) or huang hua cai in Chinese. Its flower has three petals and three sepals arranged in two layers, six stamens and one pistil, is consumed as a vegetable and used medicinally, packed with various beneficial secondary metabolites (Liu et al., 2020; Qing et al., 2021; Lv and Guo, 2023; Gao and Xie, 2023). Each flower blooms at dawn and withers by night, lasting between 9 to 17 hours, though the plant produces many buds that bloom continuously for about a month (Ma et al., 2018; Misiukevičius et al., 2023). In recent decades, research on daylilies has focused on horticultural breeding, the analysis of bioactive components, and the exploration of novel genes through high-throughput sequencing (Hou et al., 2017; Huang et al., 2022; Gao and Xie, 2023; Misiukevičius et al., 2023; Lv and Guo, 2023). A recent study suggested that tetraploid daylilies perform better than diploid varieties under arid conditions due to their enhanced adaptability and resilience to water deficit (Misiukevičius et al., 2024). *De novo* transcriptomic analysis of *Hemerocallis fulva* has been conducted to identify drought-responsive unigenes involved in hormone signaling (ABA, JA, CK, and GA) and defense responses (Cai et al., 2023).

While stomatal responses to ABA have been well-characterized in leaves, the regulation of floral stomata remains poorly understood despite their critical role in maintaining water balance during reproduction. We hypothesized that the stomatal response of daylily petals to ABA differs from that of leaves, potentially reflecting tissue-specific physiological roles and microenvironmental conditions. Using gas exchange measurements and stomatal aperture assays under controlled ABA treatments, we investigated this hypothesis to uncover fundamental features of floral stomatal physiology that may inform strategies for improving water-use efficiency in ornamental species.

## 2 Materials and methods

### 2.1 Plant materials and growth conditions

**Figure 1:**
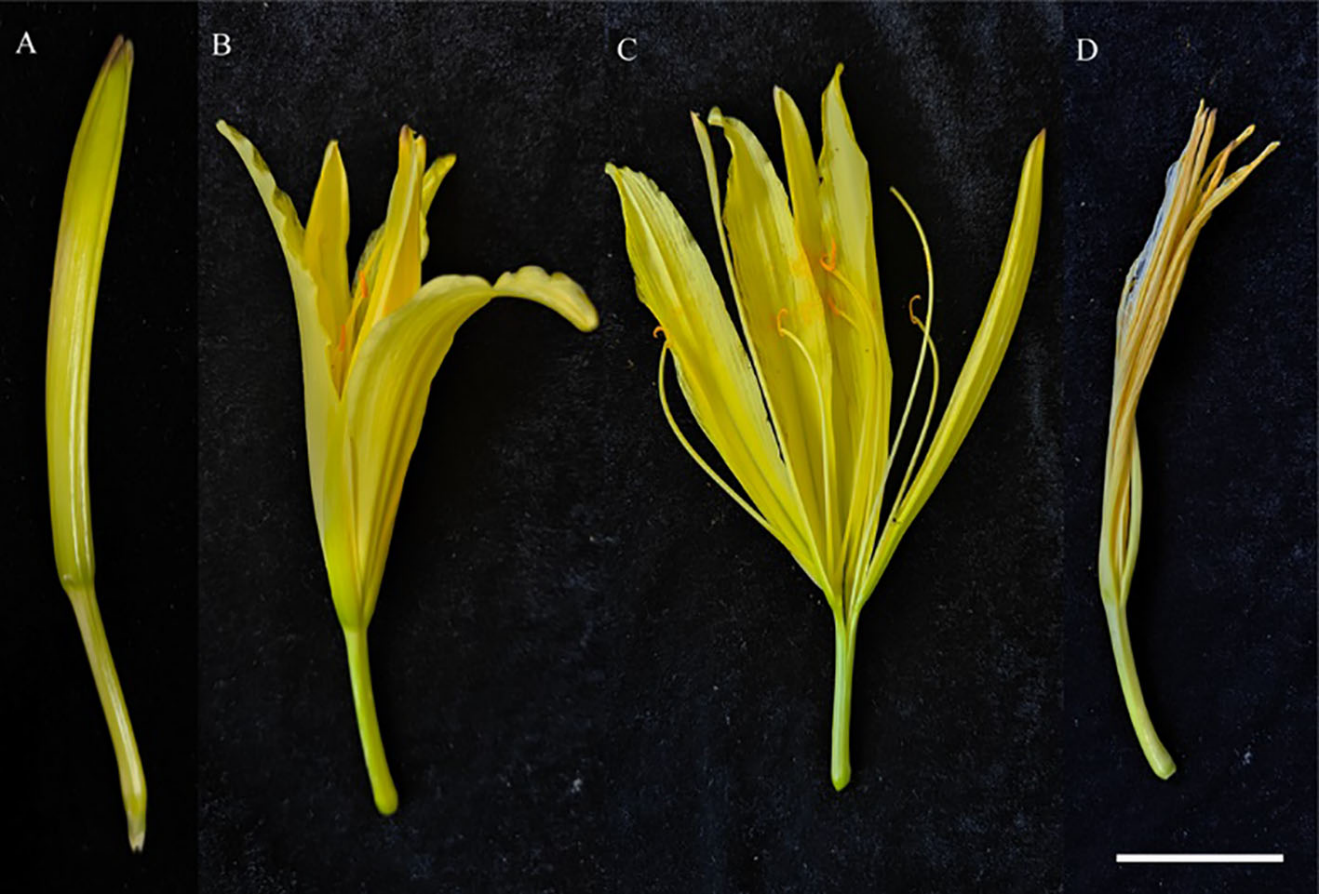
Different developmental stages of ‘Ma Lin’ flowers. **(A)** is commercial flower bud stage, **(B)** is initial flowering stage, **(C)** is late flowering stage and **(D)** is senescent stage, respectively (bar = 2 cm).

Daylilies of the ‘Ma Lin’ variety were used in this study, which have been grown from tubers with buds since 2019. The plants were maintained in the germplasm nursery at the Biological Agriculture Plantation of Longdong University, Qingyang, Gansu, China (35°43’48” N, 107°41’2” E; altitude 1480 m). The plantation is located on the Loess Plateau, which has a semi-arid temperate continental climate with an average yearly temperature of 10°C and precipitation of 600 mm. The plants in the plantation were irrigated weekly with half-strength Hoagland’s solution. The experiment was conducted in July 2023 during the flowering stage. We divided the flowering process of ‘Ma Lin’ into four stages: commercial flower bud stage, initial flowering stage, late flowering stage, and senescent stage (**Figure 1**). In addition to the diurnal variation in stomatal aperture, other indicators were determined by selecting the young petals that had just opened, specifically the petals from the initial flowering stage (**Figure 1**).

### 2.2 Stomatal morphology

Prepared epidermal strips of ‘Ma Lin’ petals in initial flowering stage (**Figure 1B**) were peeled off, and cut into small pieces of 0.5 cm × 0.5 cm. The morphology of stomata on the upper and lower epidermis, including stomata type, stomatal distribution, stomatal length, stomatal density, and distal axial ratio was observed under an optical microscope (Olympus CX21, Japan). The experiments were independently repeated each time, 30 stomata were measured for each independent replicate in each individual plant (three different plants per treatment). Mean values and standard errors were calculated from three independent replicates (total stomatal number per treatment = 90, *n* = 3). Stomatal length (μm) was defined as the distance between the junction points of guard cells. Stomatal width (μm) represented the aperture width, measured as the maximum distance between guard cells. Stomatal density (mm^-^²) was quantified as the number of stomata per unit area. The formula is stomatal density (mm^-2^) = N/A, where N represents the number of pores in the visual field and A represents the area of the visual field. Distal axial ratio (%): for two-sides stomatal plants, the distal axial ratio is the ratio of the number of stomata in the lower epidermis to the total number of stomata on the two surfaces of the petals.

### 2.3 Stomatal conductance measurement

The stomatal conductance (g_s_) was measured following a previously described method by Brodribb and McAdam (2011). For responses to ABA, pedicels were severed from 8-week-old individuals of ‘Ma Lin’ daylily, recut under distilled water, and the shoot/stem base immersed in a vial of distilled water. This prevents air from entering and forming embolism. Hydraulic properties and gas exchange are not affected by cutting the pedicel in short term (Brodribb and McAdam, 2011; Sack and Scoffoni, 2012). The g_s_ was measured immediately using an infrared gas analyzer (Li-6800, Li-Cor, Lincoln, NE, USA) with a leaf cuvette temperature of 22°C, vapor pressure deficit of about 1.2 kPa, a CO_2_ concentration of 400 µmol mol^–1^, and PPFD of 1000 µmol quanta m^–2^ s^–1^. After 10 min for equilibration in the chamber, the stomatal dynamics of the petal were measured for at least 50 min to ensure that a stable g_s_ had been reached, after which the distilled water was replaced by 50 µM ABA so that the ABA entered into the transpiration stream of the excised stem, and the g_s_ was measured until it was stable. After measurement, sample was removed from the cuvette and the petal was scanned by scanner (Epson Perfection V600 Photo Scanner, Seiko Epson Corporation, Nagano, Japan) and petal area was measured using ImageJ software (https://imagej.nih.gov/ij/) so that the g_s_ data could be adjusted for the petal area in the cuvette.

### 2.4 Stomatal aperture assay

Stomatal aperture assay of epidermal strips was carried out according to method by Pei et al. (2000). The lower (abaxial) epidermis of ‘Ma Lin’ petals was peeled with forceps, as quickly as possible without crushing the epidermis. The peeled epidermal strips were cut into tiny pieces about 0.5 cm × 0.5 cm. Epidermal strips were incubated in a petri dish containing opening buffer [10 mM 2-Morpholino ethane sulfonic acid (MES), 50 mM KCl, 20 µM CaCl_2_, pH 6.15] and exposed to light for 2 h (white LED, PPFD of 100 µmol m^–2^ s^–1^). In order to test the effect of ABA on stomata, a various concentration of ABA was added to the opening buffer with 0.1 µM, 1 µM, 10 µM, and 20 µM for 1 h, respectively. In order to explore the role of several signal substances on ABA-mediated stomatal closure, and understand the relationship between them, the peels were incubated in the corresponding inhibitors for 30 min prior to treatments with 10 µM ABA, 100 µM H_2_O_2_ or 100 µM NO donor sodium nitroprusside (SNP), the inhibitors consist of 100 U ml^-1^ H_2_O_2_ scavenging-catalase (CAT) or 200 µM NO scavenger 2-(4-carboxyphenyl)-4,4,5,5-tetramethylimidazoline-1-oxyl-3-oxide (cPTIO). Ultimately, stomatal aperture was recorded after a further six treatments for 1 h, including 10 µM ABA, 100 µM H_2_O_2_, 100 µM SNP, 10 µM ABA combined with 100 U ml^-1^ CAT, 10 µM ABA combined with 200 µM cPTIO, 100 µM H_2_O_2_ combined with 200 µM cPTIO or 100 µM SNP combined with 100 U ml^-1^ CAT. All experiments were conducted under the same environmental conditions (temperature, humidity, vapor pressure deficit). Stomatal aperture was recorded by optical microscopy with 40× objective (Olympus CX21, JAPAN). The stomatal aperture was analyzed by the image J software (https://imagej.nih.gov/ij/). First, we took a photo of the slide with the standard ruler, whose minimum scale is 10 micrometers, all parameters were the same as those of the stomatal photos. Then, the scale was set in ImageJ, a line segment of known length was drawn with the line tool, then inputted then known length value, checked Global option, and confirm. Finally, the stomatal aperture can be directly measured. When measuring stomatal aperture, 5 fields were randomly selected for each epidermal strip, 6 stomata were randomly selected for each field. 30 stomata were measured aperture for each independent replicate in each individual plant, and the mean values and standard errors of stomatal aperture were calculated by averaging across three replicates for every treatment (three different plants per treatment, the total stomatal number = 90, *n* = 3).

### 2.5 Measurement of water loss rate of petals in daylily

In order to study the effect of ABA on stomata, we measured the water loss rate of ‘Ma Lin’ petals in initial flowering stage. The experimental method was based on the method used by Liu et al. (2022) with some modifications. Flowers with a short stalk were inserted in deionized water (MilliQ, Millipore, Billerica, MA, USA) or 50 µM ABA solution as control and treatment, respectively. After 1 h of treatment in light (white LED, PPFD of 100 µmol m^–2^ s^–1^), the petals were removed, quickly dried with absorbent paper under light, and weighed at 30-min intervals for 2.5 h. Based on three independent replicates per treatment, the mean values and standard errors of the water loss rate were computed.

### 2.6 Statistical analysis

The experimental data were analyzed using SPSS 22.0 software through one-way ANOVA with Duncan’s multiple range test. Assumptions of normality and homogeneity were tested before ANOVA. A *p*-value of less than 0.05 was considered statistically significant, and distinct letters positioned above the columns in the figures indicate significant differences between control and treatment groups. Charts were created using SigmaPlot software (SigmaPlot 12.5, Systat).

## 3 Results

### 3.1 Morphological traits of stomata in daylily petals

**Figure 2:**
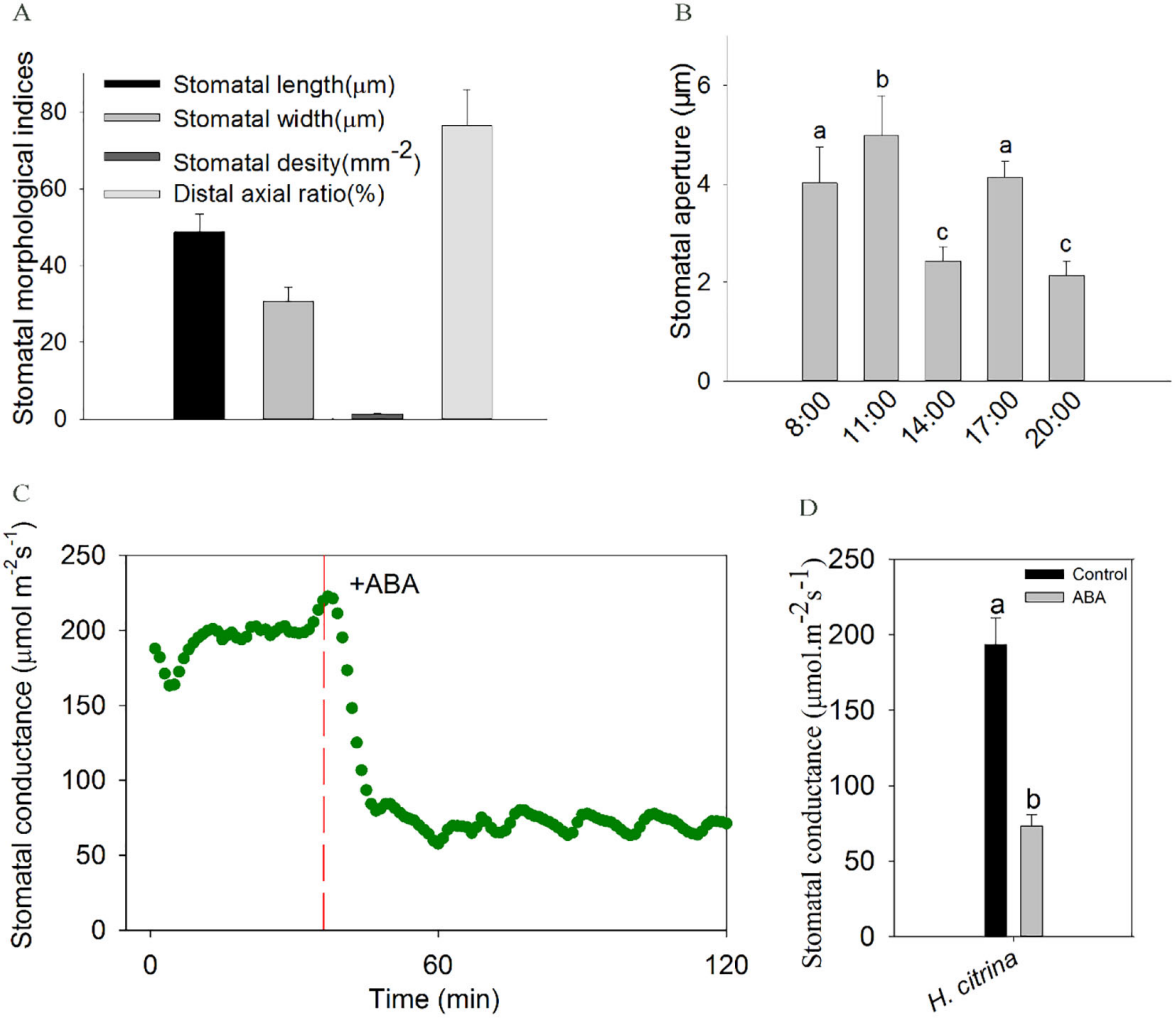
**(A)** Morphometric indices of petal stomata in ‘Ma Lin’ daylily (mean ± SD). **(B)** Diurnal variation of stomatal aperture in ‘Ma Lin’ daylily petals at five time points, with data shown as means ± SE (total stomatal number per treatment = 30, *n* = 3) and significant differences marked by different letters (*P* < 0.05); **(C, D)** Responses of stomata to exogenous ABA application on ‘Ma Lin’ daylily petals, depicting **(C)** dynamic changes in stomatal conductance over time and **(D)** average stomatal conductance before and after ABA application, with critical moments highlighted by vertical long-dash red line. Data points represent means ± SE (*n* = 3) from three individual plants, significant differences marked by different letters (*P* < 0.05).

Microscopic observations revealed that stomata were primarily distributed on the lower epidermis of the petals, accounting for 76.5% of the total stomata present on both surfaces. The stomata were arranged in a strip near the central main vein of the petals. The guard cells of the petals of daylily show reniform-shape with equal thick walls, the cell wall thickens uniformly and they were parallel type stomata according to the relationship between stomata and surrounding epidermis cells, the pores were fusiform. Unlike epidermal cells, guard cells contain a large number of chloroplasts, and the stomatal density on petals is significantly lower than that on leaves (**Figure 2A**).

### 3.2 Diurnal variation of stomatal aperture

The daylily flower usually opens in the morning and withers away in the evening. We investigated diurnal changes in stomatal aperture during the process of flower opening to flower closing throughout the day. There are two peaks at 11:00 and 17:00, and a trough at 14:00, this may be due to the biological clock causing a marked decrease in stomatal aperture at 14:00 due to the photosynthetic noon break with excessive light intensity, high temperatures. The stomatal aperture reaches a lower value at 8 PM (**Figure 2B**).

### 3.3 Petal stomata of daylily are sensitive to ABA

**Figure 3:**
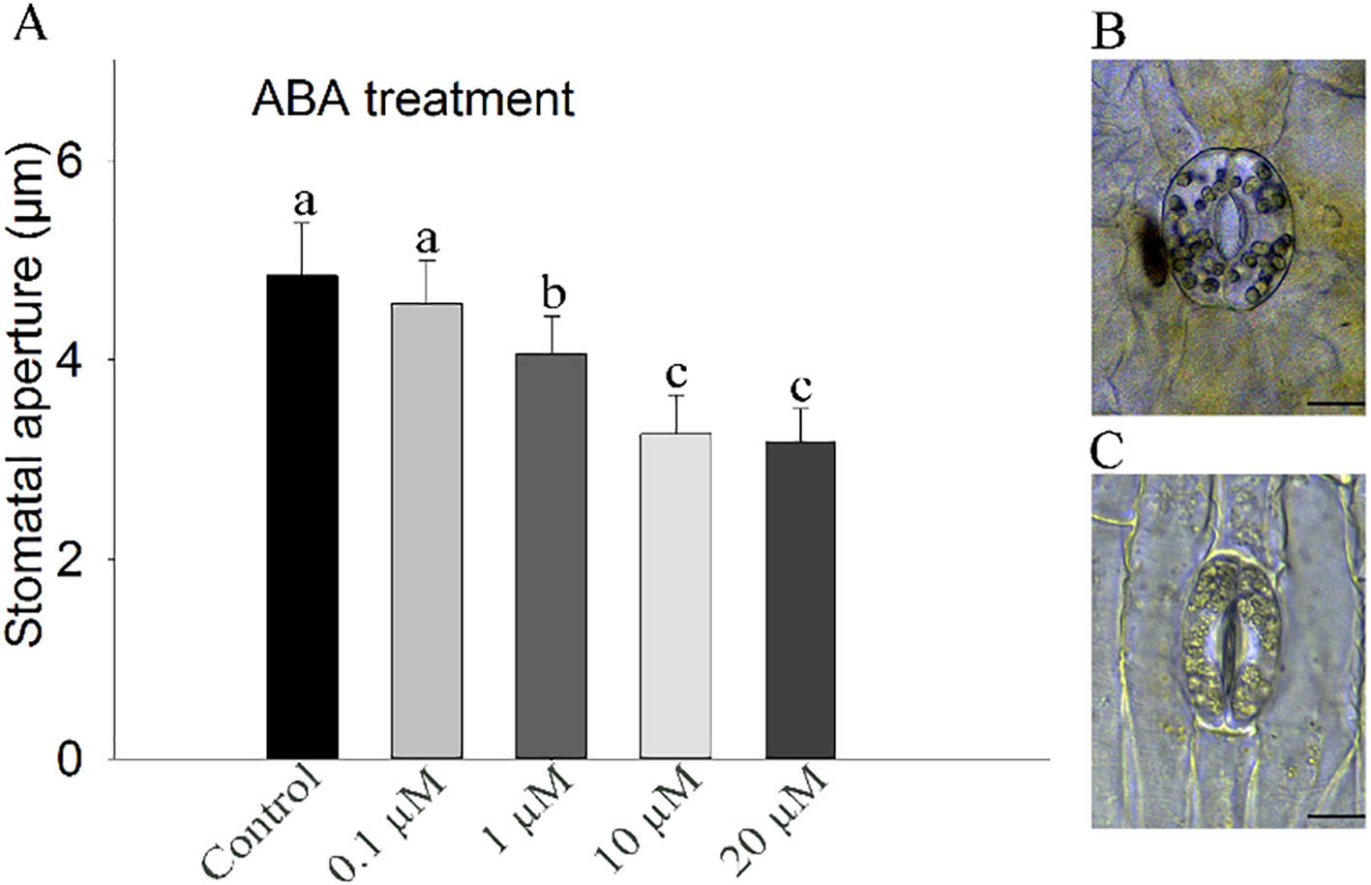
Stomatal aperture of ‘Ma Lin’ daylily petals in response to different concentrations of ABA. **(A)** Stomatal responses to varying concentrations of ABA. **(B, C)** Images of epidermal peels showing stomatal apertures before and after ABA treatment, respectively (bar = 10 µm). The presented data represent means ± standard error (SE), with a total of 30 stomata counted per treatment and three replicates (*n* = 3). Different letters indicate significant differences between treatments (one-way ANOVA, *P* < 0.05).

To explore the physiological responses of stomata to ABA in floral organ of daylily, petal gas exchange was measured. The result showed exogenous ABA induced a rapid reduction in g_s_ in the daylily petals (**Figures 2C, D**). Meanwhile, stomatal aperture assay was conducted, after exogenous ABA treatments at different concentrations, the results showed that the stomata of petal epidermal strips were sensitive to exogenous ABA, and there was a concentration gradient effect, and stomatal aperture gradually decreased with the increase of concentration. The stomatal aperture of the petals of daylily decreased by 32.78% after 10 µM ABA treatment for 1 h, the difference is significant (*P*<0.05) (**Figure 3**). Since the effect of 10 μM ABA on stomatal closure was found to be similar in magnitude to the effect of 20 μM ABA, which is known to have toxic effects and lead to tissue necrosis (Cardoso and McAdam, 2019), we selected 10 μM ABA for the subsequent stomatal aperture experiment.

**Figure 4:**
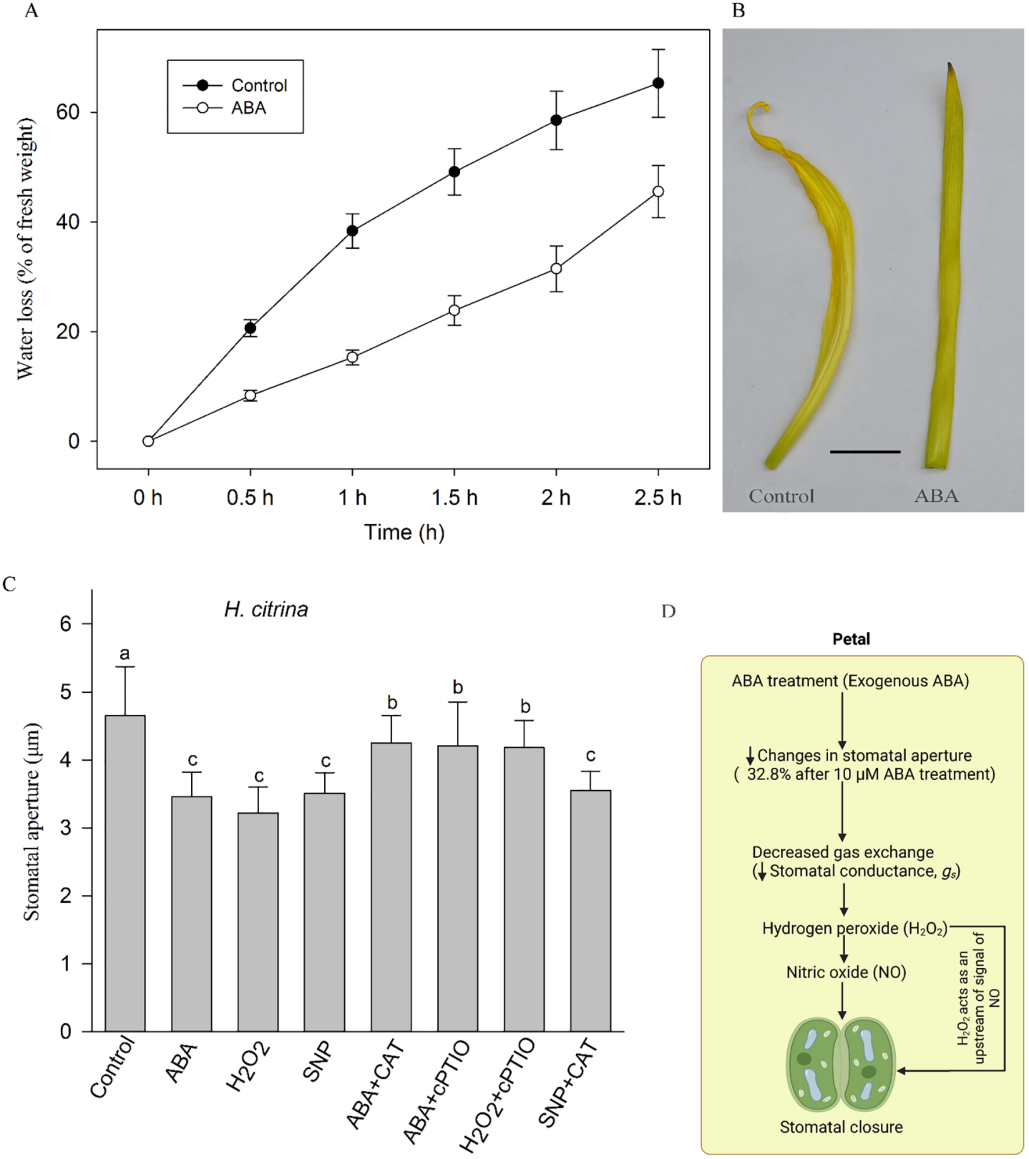
Effects of ABA on water loss in ‘Ma Lin’ daylily petals: **(A)** Time-course dynamics of water loss rate in petals after treatment with ionized water and ABA (*n* = 3). **(B)** Image of isolated petals after treatment of 2.5 h under light between deionized water treatment and 50 µM ABA treatment, respectively (bar = 1 cm). **(C)** Stomatal responses to various ABA signaling substances for 1 h, showing mean values ± SE with each treatment involving a count of 90 stomata across three replicates (*n* = 3). ABA, abscisic acid; H_2_O_2_, hydrogen peroxide; CAT, H_2_O_2_ scavenging-catalase; cPTIO, NO scavenger 2-(4-carboxyphenyl)-4,4,5,5-tetramethylimidazoline-1-oxyl-3-oxide; lowercase letters denote statistically significant differences between treatments and the control group (one-way ANOVA, *P* < 0.05). **(D)** Conceptual model of ABA-mediated signaling in the petals of ‘Ma Lin’ daylily. Exogenous application of abscisic acid (ABA) results in a significant reduction in stomatal aperture (32.8% decrease following 10 µM ABA treatment), leading to reduced stomatal conductance (g_s​_). The proposed pathway suggests that hydrogen peroxide (H_2_O_2_) acts upstream of nitric oxide (NO) in mediating the ABA-induced stomatal closure in floral tissues. This model summarizes key findings from the current study and integrates them with relevant insights from existing literature, highlighting a possible H_2_O_2_–NO signaling hierarchy specific to petal stomatal regulation.

In addition, we measured the water loss rate of isolated petals in daylily plants, it was found that after exogenous ABA treatment for 1 h, the water loss rate of petals of daylily was significantly lower compared with that of control (*n* = 3, *P*<0.05) (**Figure 4A**). The surface of the petals treated in deionized water became more crumpled than those treated in ABA solution (**Figure 4B**).

### 3.4 Signaling on ABA induced stomatal closure

In order to further explore the signal transduction process of ABA-induced stomatal closure, we investigated the effect of exogenous ABA, H_2_O_2_ and NO donor SNP and corresponding scavengers on stomatal closure in petals of ‘Ma Lin’. The experimental results show that ABA and its downstream signaling pathway substances, such as H_2_O_2_, SNP, can cause stomatal closure. ABA-induced stomatal closure is inhibited by peroxide scavenger enzymes (CAT) and nitric oxide scavenger (cPTIO). Stomatal closure induced by hydrogen peroxide is also inhibited by cPTIO, but NO induced stomatal closure could not be inhibited by CAT (**Figure 4C**).

## 4 Discussion

### 4.1 What is the function of petal stomata of daylily?

Stomata in vascular plants are central to regulating gas exchange and water loss, primarily through leaf tissues (Wexler et al., 2024; Nasr Esfahani and Sonnewald, 2024). In contrast, the role of stomata on floral organs remains less understood and varies across species (Gupta et al., 2020). In the petals of *Hemerocallis citrina* (‘Ma Lin’), stomata are distributed predominantly along the lower epidermis, particularly near the central vein. These stomata possess guard cells rich in chloroplasts, distinguishing them from the surrounding petal cells, which are largely devoid of chloroplasts (**Figures 3B, C**). The functional significance of the abundance of chloroplasts in the guard cells of expanded mature petals remains to be explored in the future.

Stomata regulate gas exchange, mainly facilitating water vapor loss and CO_2_ uptake. However, stomata occupy a small proportion in petal tissue, the contribution of guard cells to photosynthesis is minimal, there are almost no chloroplasts in the petal mesophyll and epidermal cells, so do the stomata open only for transpiration? Flowering represents a pivotal developmental transition from the vegetative stage to the reproductive stage. The timing of flowering must be carefully controlled under ever-changing environmental conditions to ensure successful reproduction. Extensive genetic and molecular biological analyses in the model species *Arabidopsis thaliana* have identified important regulators of flowering time and revealed a complex network of highly interconnected pathways that are regulated by seasonal cues (e.g., light and temperature) and internal factors (e.g. age and nutrient availability) (Susila et al., 2016). Thus, we assume that important role of petal stomata may be related to reproductive processes, such as helping to distribute aromas to attract pollinators (Li et al., 2020). Recent research found that petal transpiration elevated humidity in the flower headspace of two flower species (Harrap and Rands, 2022), which indicated stomata influencing humidity and temperature conditions in the flower’s internal microenvironment, thereby indirectly supporting pollination and seed formation. Meanwhile, more stomata were found along the petal vein, stomatal aperture is small and density is lower. During flowering, high turgor pressure is required to maintain the shape of the petals, which necessitates limited water loss through transpiration, potentially explaining these stomatal features.

The stomatal aperture of ‘Ma Lin’ petals shows a fluctuant trend throughout the day. Stomatal opening and closing are strictly affected by environmental factors, such as temperature and light (Manandhar et al., 2024). At 8 AM, the temperature is lower, the light is weaker, and the stomata are not fully open. As the light intensity and temperature increase, the stomatal aperture reaches its maximum around 11 o’clock. Around 2 PM., there was a midday depression phenomenon, so the stomatal aperture was greatly reduced, then the flowers gradually close and shrink, and the stomata gradually close. Thus, our results on daily fluctuation in stomatal aperture suggest that the trends in stomatal aperture resembles the pattern and logic that is known to occur in leaf stomata.

### 4.2 The petal stomata of ‘Ma Lin’ are sensitive to ABA

In vascular plants, stomatal closure has both active and passive modes. The exact point in the evolution of land plants, at which the active regulation of the function of leaf stomata first appeared, has been a matter of extensive debate. The view of gradualistic evolution suggests that stomatal control of leaf water balance under drought stress was achieved through the accumulation and active regulation of plant hormone ABA in seed plants, while stomata did not respond to ABA in ferns (Brodribb and McAdam, 2011; McAdam and Brodribb, 2012). However, the stomatal response of flower to exogenous ABA is still unclear. Our study demonstrates that exogenous ABA application induces a dose-dependent reduction in stomatal aperture and gas exchange in ‘Ma Lin’ petals. Specifically, a 10 μM ABA treatment led to a ~33% decrease in stomatal aperture. Moreover, ABA-treated petals exhibited a significantly reduced rate of water loss (**Figures 4A, B**), supporting the presence of active hormonal regulation. These results provide clear evidence that daylily petal stomata are responsive to ABA, suggesting that floral tissues, like leaves, possess physiologically functional stomatal control mechanisms that may contribute to water-use efficiency and stress adaptation during reproduction.

### 4.3 Signaling pathway accounting for stomatal responses to ABA

In the past decade, researchers are interested in the stomatal function of different plant lineages. Specifically, the divergence in the emergence and regulation of active stomatal control has been a key focus in recent evolutionary studies (Chater et al., 2011; Cai et al., 2017; Gong et al., 2021). Under drought stress, ABA is rapidly synthesized in angiosperm leaves, which subsequently triggers the production of hydrogen peroxide and nitric oxide. Concurrently, ABA activates ion channels and aquaporin activity in the guard cell membrane, ultimately leading to stomatal closure (Raghavendra et al., 2010). To investigate the signal components of ABA-induced stomatal closure signal transduction pathway in petal stomata of ‘Ma Lin’ daylily and the relationship between them, the stomatal response was measured by pharmacological experiments. The results showed that both H_2_O_2_ and NO were involved in the process of stomatal closure induced by ABA, and H_2_O_2_ performs function as an upstream component of NO, which is consistent with ABA signaling pathway of stomata in leaves (Srivastava et al., 2009). This suggests that the signaling pathway of ABA-induced stomatal closure may have evolved simultaneously in different organs of angiosperms, that means both flowers and leaves may have developed efficient active stomatal control mode that can adapt to drought during the evolution of stomata function, which might explain the wide distribution of ‘Ma Lin’ daylily in arid areas to some extent.

During the growth and development of plants, they encounter various biotic and abiotic stresses, and organisms have evolved multiple signaling pathways to cope with the corresponding environmental stresses (Zhu, 2016). The signaling pathway is not a simple linear feature, but an intricate signaling web with extensive cross-talk (Hiyama et al., 2017). Sometimes different external stimuli will induce the same signaling pathway and produce the same secondary messenger, such as hydrogen peroxide and nitric oxide. Therefore, we speculate that the stomata of ‘Ma Lin’ also has the active metabolic regulation of water balance under other stresses. Although individual flower of ‘Ma Lin’ daylily only last for about a day, there are many buds on a single plant, and it can continue to bloom dozens of flowers within a month. During the flowering process, petals need more water to maintain turgor pressure, so when subjected to mild drought, petals need to quickly close stomata to reduce water loss. In severe drought, ‘Ma Lin’ daylily will delay its flowering time. However, the regulation of stomata is diverse, and flowers of a larger number of angiosperm species need to be investigated to confirm ABA sensitivity. Our findings offer valuable insights into floral stomatal arrangement and ABA responsiveness. While we discuss broader themes such as plant adaptation and signaling pathways, these were not directly studied and are suggested as future directions. Comparative studies on leaf and flower stomatal responses would further enhance understanding of tissue-specific adaptation and ABA regulation, with implications for horticultural and agricultural applications.

### 4.4 Consequences and Implications of research

This study highlights the key role of abscisic acid (ABA) in inducing stomatal closure in daylily petals, offering insights with multiple implications. (a) Evolutionary significance: The findings contribute to a better understanding of stomatal evolution and function in petals and sepals. (b) Horticultural application: The results suggest a practical approach for extending the vase life of cut flowers and delaying senescence by reducing water loss through ABA application, which could enhance horticultural value and economic returns. (c) Taxonomic utility: The variation in epidermal morphology observed in this study may serve as a useful diagnostic tool for species identification in daylilies. (d) Agronomic relevance: Since ABA plays a central role in drought resistance, our findings provide a theoretical basis for improving water and fertilizer management during daylily cultivation under water-limited conditions.

## Data Availability

The original contributions presented in the study are included in the article/supplementary material. Further inquiries can be directed to the corresponding authors.
